# Human Parainfluenza Virus Type 3 Matrix Protein Reduces Viral RNA Synthesis of HPIV3 by Regulating Inclusion Body Formation

**DOI:** 10.3390/v10030125

**Published:** 2018-03-11

**Authors:** Shengwei Zhang, Qi Cheng, Chenxi Luo, Yali Qin, Mingzhou Chen

**Affiliations:** 1 State Key Laboratory of Virology and Modern Virology Research Center, College of Life Sciences, Wuhan University, Wuhan 430000, China; zsw189@163.com (S.Z.); qicheng@whu.edu.cn (Q.C.); luochenxiky@126.com (C.L.); 2 Hubei University of Chinese Medicine, School of Laboratory Medicine, Wuhan 430000, China

**Keywords:** human parainfluenza virus type 3, viral replication, virus-like particles, inclusion body formation, M–N interaction

## Abstract

Human parainfluenza virus type 3 is one of the main causes of lower respiratory illness in newborns and infants. The role of the matrix protein (M) in viral budding is extensively studied, but the effect of M on viral replication remains to be determined. Using an HPIV3 minigenome assay, we found that M reduced HPIV3 mingenome-encoded reporter activity even though it had an unspecific effect on the expression of cellular genes. Furthermore, the inhibition effect of M on viral RNA synthesis was proven to be independent of its virus-like particles (VLPs)’ release ability. A VLP’s defective mutant (M_L302A_) decreased the expression of minigenome reporter as wild type M did. Using an immunofluorescence assay, we found that M weakened the formation of inclusion bodies (IBs), although it did not co-localize with the IBs. Moreover, using another mutant, M_L305A_ , which is defective in M-nucleoprotein (N) interaction, we found that M_L305A_ had no effect on reporter activity and IB formation as the wild type of M did. Taken together, we conclude that M reduces the replication of HPIV3 and IB formation by M–N interaction.

## 1. Introduction

*Human Parainfluenza Virus Type* 3 (HPIV3), a non-segmented, negative-strand RNA virus (NNSV), causes lower respiratory illness in newborns and infants [1]. The genome of HPIV3 encodes at least six proteins: the nucleoprotein (N), phosphoprotein (P), RNA-dependent RNA polymerase (L), matrix protein (M), and two virus–host cell interaction glycoproteins, fusion protein (F) and hemagglutinin-neuraminidase (HN). N forms an N-RNA template that associates with P and L to form a ribonucleoprotein (RNP) complex for transcription and replication [2]. Genes in the HPIV3 genome, which is composed of gene start (GS), gene’s Open reading frame (ORF) and gene end (GE) are separated by conserved gene junction (GJ). Although sequences in GJ cannot be translated, they play a pivotal role in genes’ transcription. GS and GE, located at the beginning and end of each gene, are used as transcriptional start and polyadenylation stop signals, respectively [3]. 

N can be divided into a conserved N-terminal moiety (N_core_) and a disordered C-terminal moiety (N_tail_) [4]. N_core_ is responsible for N self-assembly and RNA-binding [5,6]. N_tail_ is indispensable for the N-RNA complex to bind to P [4]. P is a significant cofactor for L to bind to the N-RNA template. The C terminal of P mediates the association between the L and the N-RNA template and plays an important role in RNP complex formation [7]. On the other hand, the N terminal of P binds to N and keeps N soluble [8]. The interaction between HPIV3 N and P provides the minimal requirement for the formation of inclusion bodies (IBs). IBs are regarded as viral replication centers in which viral RNAs are synthesized [9]. Our previous results show that the formation of IB is a kinetic process in which small IBs fuse with each other and form large IBs where viral RNA synthesis preferentially occurs [10].

M of NNSVs are a multifunctional protein that is crucial in directing viral assembly and budding. A lack of M dramatically impairs viral release [11,12]. Previous studies have shown that M of most NNSVs has the ability to release virus-like particles (VLPs) by itself or with the help of other viral proteins, which can be used to study the mechanisms of viral assembly and budding [13]. M of *vesicular stomatitis virus* (VSV) [14], *respiratory syncytial virus* (RSV) [15], *Nipah virus* (NiV) [16], *Ebola virus* (EBOV) [17] and *measles virus* (MeV) [18] are sufficient to form and release VLPs by themselves. M of parainfluenza virus type 5 (PIV5) [19] and mumps virus [20] needs the help of N and F or HN to release VLPs. Our previous data demonstrated that HPIV3 M could release VLPs [21]. M of HPIV3 could package the N–P complex into VLPs [22]. In addition, M also plays an important role in escaping innate immunity via multiple and synergistic strategies. 

Previous studies showed that M could regulate viral replication and/or transcription in many NNSVs. Purified M of VSV inhibited viral transcription in vitro by condensing the nucleocapsid into a tight structure [23]. M of RABV shifts viral RNA synthesis from transcription to replication [24]. MeV M inhibits viral RNA synthesis and assembly by retaining the RNP complex at the plasma membrane [25]. VP40 and VP24 of EBOV are also involved in inhibiting the RNA synthesis [26]. Similarly, we found that HPIV3 M also inhibits viral replication. However, the underlying mechanism is not clear. As viral replicaiton occurs in IBs, it will be of interest to explore whether HPIV3 M inhibits viral replication and the formation of IBs. It was reported that RSV M localized into IBs via the interaction between M and M2-1 protein. M association with IBs was regarded as a potential switch between replication and transcription [27]. Because M of HPIV3 interacts with N and P, we sought to know whether HPIV3 M inhibits the formation of IBs and viral replication. 

In this study, we present evidence that HPIV3 M inhibits viral replication and the inhibitory effect is independent of its VLP formation function. Importantly, using an M mutant (M_L305A_), which is defective in M–N interaction, we demonstrate that M inhibits HPIV3 replication and IB formation via its interaction with N.

## 2. Materials and Methods 

### 2.1. Cells and Virus

HeLa and LLC-MK_2_ cells were maintained in Dulbecco’s modified Eagle’s medium (DMEM) with 8% fetal bovine serum (FBS). 293T-T7 cells were propagated in DMEM containing 20% FBS and 1 μg/mL puromycin. *Recombinant vaccinia virus* (vTF7-3) expressing bacteriophage T7 RNA polymerase was propagated in HeLa cells at a multiplicity of infection (MOI) of 0.1. HPIV3 (NIH 47885), HPIV3_MGFP_ and HPIV3_HA-P_ were propagated in LLC-MK_2_ cells by inoculating at an MOI of 0.1. 

### 2.2. Plasmid Constructs

The plasmids pGEM4-N, pGEM4-P, pGEM4-L, pOCUS-HPIV3-MG, pOCUS-HPIV3, pCAGGS-M, pCAGGS-M_L302A_ and pCAGGS-M_L305A_, which encode untagged N, P, L, HPIV3 minigenome, HPIV3 genome, M and M mutants have been described previously [9,21,22]. cDNAs encoding M mutants were amplified by PCR-based cloning techniques using pCAGGS-M as a template. PCR products were cloned into pcDNA3.0 (for T7 promoter-driven protein expression). cDNA encoding renilla luciferase was amplified using pRL-TK as a template and the PCR product was cloned into pGBKT7. The PCR-based approach was also applied to construct HPIV3_MGFP_ genome as described elsewhere [9]. PCR products of GFP were inserted into the gene end of M by overlapped PCR. The PCR product was cloned into pOCUS-HPIV3 digested by SalI and Eco91I via ligase independent cloning. All constructs were verified by DNA sequencing.

### 2.3. In Vitro Minigenome Assay of HPIV3

The in vitro HPIV3 minigenome assay was performed following the procedure described by Hoffman and Banerjee [3]. HeLa cells in 12-well-plates were infected with vTF7-3 at an MOI of 1 for 1 h. pGEM4-N (125 ng), pGEM4-P (62.5 ng), pGEM4-L (100 ng) and pOCUS-HPIV3-MG (50 ng) were transfected alone or together with pcDNA3.0-HA-M or M mutants by Lipofectamine 2000 (Invitrogen, Carlsbad, CA, USA). In addition, 5 h post-transfection (pt), the transfection medium was replaced by DMEM with 4% FBS. In addition, 24 h pt, the medium was removed and cells were harvested. Furthermore, 150 µL of lysis buffer were used to lyse the cells and an aliquot of 20 µL was used for luciferase activity analysis according to the manufacturer’s instructions. All assays were repeated at least three times for accuracy.

### 2.4. RNA Extraction and RT-PCR

RNA extraction was carried out by using TRIZOL reagent (ambion, Invitrogen) according to the manufacturer’s instructions. RNAs were dissolved in 40 µL RNase-free water. In addition, 1 µg RNA of each sample was used for reverse transcription (RT) using a reverse transcriptase (Fermentas, Waltham, MA, USA) following the manufacturer’s instructions. Oligo (dT) was used for RT and the primer pairs 5′-GGATGTCTTCTTACTCGGCT-TC-3′, 5′-CATTTCTTTCGCTTTG-ACTGTTCT-3′ and 5′-CAAGCAG-AAGAACGGCATCAAG-3′, 5′-TCA-CGAACTCCAGCAGGACCATG-3′ were used for PCR to amplify the cDNA of M and GFP, respectively.

### 2.5. Immunofluorescence Assay

HeLa cells grown on coverslips in 24-well-plates were transfected with 0.5 µg pCAGGS-P, 0.125 µg pCAGGS-N and increasing amounts of pCAGGS-M or 0.5 µg M mutants by Lipofectamine 2000 (Thermo Fisher Scientific, Waltham, MA, USA). 24 h pt, cells were washed with cold PBS and fixed with 4% paraformaldehyde for 20 min. Then, the cells were permeabilised with 0.2% Triton X-100 for 20 min at room temperature. Blocking the permeated cells by 3% bovine serum albumin (BSA) in PBS for 0.5 h at room temperature and incubating the cells with mouse monoclonal anti-HA antibody (Sigma, St. Louis, MI, USA; 1:2000) and rabbit polyclonal anti-Myc antibody (Santa Cruz, CA, USA; 1:200) in 1% BSA at room temperature for 2 h. Then, the cells were washed with 1% BSA in PBS and incubated with the goat anti-mouse IgG fluorescein (Thermo; 1:200) and goat anti-rabbit IgG rhodamine (Thermo; 1:100) secondary antibody for 2 h at room temperature. After being washed three times with 1% BSA in PBS, the coverslips were turned over and the cells were incubated with a drop of DAPI (4′,6-diamidino-2-phenylindole)-contained Fluorshield for nuclear staining. Confocal images were collected by an Olympus confocal FV1000 microscope (Olympus, Tokyo, Japan).

### 2.6. Transfection and Recovery of Recombinant HPIV3

For the experiment, 293T-T7 cells in 12-well-plates, grown to 40% confluence, were transfected with pGEM4-N (200 ng), pGEM4-P (200 ng), pGEM4-L (100 ng), and pOCUS-HPIV3 or pOCUS-HPIV3_MGFP_ (2 µg) via calcium phosphate transfection. Four days pt, the mediums were collected. The cells were harvested in another 300 µL of DMEM. After being frozen and thawed three times, the cells were subjected to centrifugation at 5000 rpm at 4 °C. The clarified supernatant was collected and mixed with the collected mediums. The mixtures were layered onto a fresh LLC-MK_2_ cell monolayer to amplify the recombinant HPIV3 at 37 °C. Four days later, the mediums were harvested and viral titers were determined. A single recovered recombinant HPIV3 clone was isolated during the titer determination and used to infect fresh LLC-MK_2_ cell for amplification.

## 3. Results

### 3.1. M Reduces HPIV3 Mingenome-Encoded Reporter Activity

**Figure 1:**
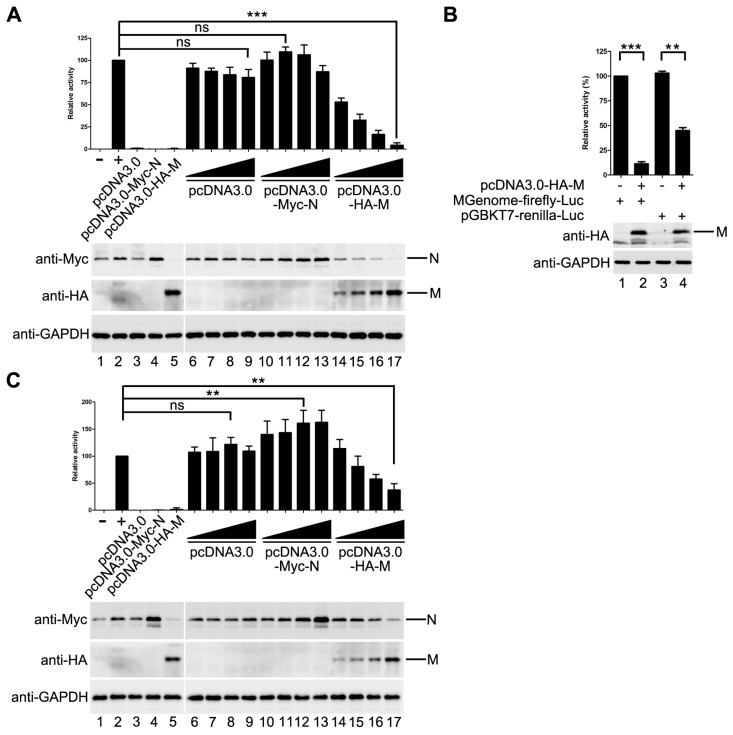
M decreases minigenome reporter activity. (**A**) the effect of M on minigenome-encoded reporter RNA synthesis. vTF7-3-infected HeLa cells were transfected with plasmids encoding N (125 ng), P (62.5 ng), L (100 ng) and minigenome (50 ng) and increasing amounts of pcDNA3.0 vector or plasmids encoding M and N (100, 200, 400, 800 ng). The amount of plasmids was kept constant by pGBKT7. Relative luciferase activity was measured according to the manufacturer’s instructions. The expression of M and N were detected via Western blot with anti-HA and anti-Myc antibody respectively. GAPDH was used as a loading control. Values are means ± SD from three experiments; Student’s *t* test: *** *p* < 0.001, ns = not significant. “+,−” means with or without transfecting P plasmid. (**B**) M has an effect on the expression of T7-promoter driven renilla luciferase. Plasmid encoding renilla luciferase (5 ng) and plasmids encoding N, P, L and minigenome were transfected alone or together with 800 ng pcDNA3.0-HA-M in vTF7-3-infected HeLa cells. Relative luciferase activity was measured and the expression of M was detected via Western blot with anti-HA antibody.Values are means ± SD from three experiments; Student’s *t* test: ** *p* < 0.01, *** *p* < 0.001, “+,−” means with or without transfecting corresponding plasmids. (**C**) M decreases minigenome-encoded reporter RNA synthesis. Plasmids encoding N (125 ng), P (62.5 ng), L (100 ng), minigenome (50 ng) and renilla luciferase (5 ng) were transfected with increasing amounts of of pcDNA3.0 vector or plasmids encoding M and N (100, 200, 400, 800 ng) in vTF7-3-infected HeLa cells. The amount of plasmids was kept constant by pGBKT7. The activities of firefly luciferase and renilla luciferase were measured according to the manufacturer’s instructions. Relative activity of firefly luciferase to renilla luciferase was calculated and the expression of M was detected via Western blot as described for (**A**). Values are means ± SD from three experiments. Student’s *t* test: ** *p* < 0.01, ns = not significant. “+,−” means with or without transfecting P plasmid.

Previous studies of some NNSVs such as VSV, MeV and EBOV showed that M inhibits viral replication and/or transcription. Thus, we sought to determine whether HPIV3 M has a similar effect on the replication of HPIV3. An HPIV3 minigenome assay was performed with increasing amounts of pcDNA3.0 vector, HA-tagged M and Myc-tagged N. The result showed that M decreased the mingenome-encoded reporter activity in a dose-dependent manner, while the pcDNA3.0 vector had no obvious effect on the reporter activity. N increased the reporter activity at its low concentration and inhibited the reporter activity at its high concentration. When 800 ng pcDNA3.0-HA-M was transfected, the activity of report gene decreased to 5% of that in M mock transfected control (Figure 1A). 

As it was reported in other NNSVs that M has an unspecific effect on the expression of cellular genes, we could not rule out the possibility that the activity inhibition of the minigenome reporter was due to the expression reduction of cellular genes including the minigenome assay components because we found that the expression of N was also decreased with the increasing amount of M (Figure 1A, upper panel, lane 14–17). 

In order to confirm the possibility mentioned above, a control plasmid that encodes renilla luciferase under the control of T7 promoter was constructed to represent the expression of the minigenome assay components. The activity of renilla luciferase was adjusted to a comparable level to that of minigenome firefly luciferase (Figure 1B, lane 1 and lane 3). Then, the plasmids expressing M were transfected with pGBKT7-renilla luciferase and the minigenome assay components, respectively. The activity of minigenome encoded firefly luciferase decreased dramatically as detected in Figure 1A, and the activity of renilla luciferase dropped to 41% of that in M mock transfected control (Figure 1B, compared lane 3 to lane 4), suggesting that HPIV3 M indeed has a negative effect on the expression of T7 promoter-driven protein expression. Thus, it is not clear whether M decreased the expression of the minigenome reporter by reducing the expression of the minigenome assay components, or it has an inhibitory effect on viral RNA synthesis. To address this issue, we modified our minigenome system by co-expressing the minigenome-encoded firefly luciferase with T7 promoter-driven renilla luciferase. The activities of firefly luciferase and renilla luciferase, reflecting minigenome-encoded reporter RNA synthesis and the expression of plasmid-encoded proteins, respectively, were measured. The ratio of firefly luciferase to renilla luciferase was calculated to reflect the direct effect of M on minigenome-encoded reporter RNA synthesis. As shown in Figure 1C, the ratio of firefly luciferase to renilla luciferase dropped gradually with increasing expression of M. When 800 ng of pcDNA3.0-HA-M was transfected, the ratio of firefly luciferase to renilla luciferase dropped to 35% of that in M mock transfected control. pcDNA3.0 vector has no effect on the expression of the minigenome reporter; N increased the expression of the minigenome reporter (Figure 1C). Taken together, these data suggested that M inhibits viral minigenome-encoded reporter RNA synthesis besides a negative effect on the expression of plasmid-encoded proteins.

### 3.2. M Restricts the Replication of HPIV3 

**Figure 2:**
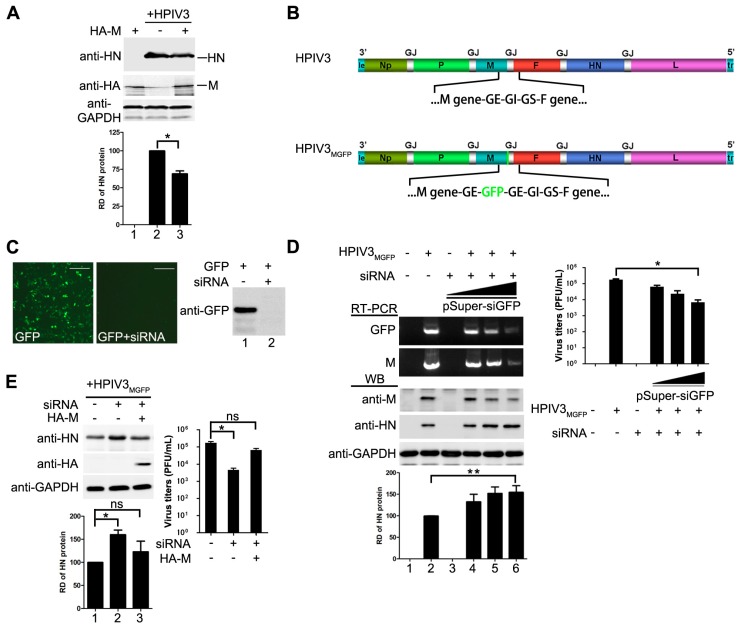
M reduces HPIV3 replication. (**A**) over-expression of M reduces the replication of HPIV3. HeLa cells were transfected with vector or plasmids encoding M (2 µg). At 24 hpt, the cells were infected with HPIV3. At 48 hpt, the cells were harvested and lysed for Western blot. Quantity one software was used to quantify the band intensities of HN in the lysates. Values are means ± standard deviations (SD) from three independent experiments. Student’s *t* test: * *p* < 0.05; “+,−” means with or without transfecting M plasmid. (**B**) genomic structure of HPIV3_GFP_ generated in this study. A GFP gene was inserted into the gene end of M and the recombinant virus was rescued as described in Materials and Methods; (**C**) validity test of pSuper-siGFP. Plasmid encoding GFP (50 ng) was transfected alone or together with 2 µg of pSuper-siGFP in HeLa cells. At 24 hpt, the cells were subjected to fluorescence detection and Western blot assay;“+,−” means with or without transfecting corresponding plasmids. Scale bar, 100 µm. (**D**) knocking down of M slightly promotes the replication of HPIV3 and extensively reduces viral budding. HeLa cells were transfected with increasing amounts of pSuper-siGFP (0.5 µg, 1 µg, 2 µg). At 24 hpt, the cells were infected with HPIV3_MGFP_. At 48 hpi, half of the cells were collected for RNA extraction. RT-PCR was used to confirm the effect of siGFP on the transcription of M-GFP mRNA. The other half of cells was lysed for Western blot to detect the expression of M and HN. Supernatants were harvested for titer determination. Quantity one software was used to quantify the band intensities of HN in the lysates. Values are means ± standard deviations (SD) from three independent experiments. Student’s *t* test: * *p* < 0.05, ** *p* < 0.01; “+” means positive transfected or infected corresponding plasmids or viruses. “−” means negative transfected or infected corresponding plasmids or viruses. (**E**) re-expression of M rescues viral titer and counteracts effect of pSuper-siGFP knockdown. HeLa cells were transfected with 2 µg of pSuper-siGFP. At 24 hpt, the cells were infected with HPIV3_MGFP_. At 24 hpi, HeLa cells were transfected with 1 µg of pcDNA3.0 vector or pcDNA3.0-HA-M. At 48 hpi, cells were harvested and lysed for Western blot assay. Supernatants were harvested for titer determination. Quantity one software was used to quantify the band intensities of HN in the lysates. Values are means ± standard deviations (SD) from three independent experiments. Student’s *t* test: * *p* < 0.05, ns = not significant. “+,−” means with or without transfecting corresponding plasmids.

Having showed that M of HPIV3 decreases viral minigenome-encoded reporter RNA synthesis, we sought to determine whether M has a similar effect on viral replication of HPIV3. HeLa cells were transfected with plasmids encoding M or empty vector and then infected with HPIV3 at 48 hpt, cells were harvested and lysed for Western blot to detect the expression of viral HN. As shown in Figure 2A, cells that were transfected with M resulted in a lower level of viral HN expression, suggesting that exogenous expression of M restricted the replication of HPIV3. Then, we sought to know whether depletion of M would cause the opposite effect. Since no valid siRNA targeting the mRNA of M is available, we rescued a recombinant HPIV3 virus, termed HPIV3_MGFP_, in which a full-length GFP gene was inserted into the genome frame after the termination codon of M and in front of the gene end (Figure 2B). Although the GFP gene in HPIV3_MGFP_ will not be expressed, it will be transcribed following the transcription of M gene that generates a read-through M-GFP mRNA until the GE sequences. Thus M can be knocked down easily via a siRNA targeted GFP mRNA. When cells were infected by HPIV3_MGFP_, it will generate intact M as wild type HPIV3 infection. Plasmid pSuper-siGFP was used to express siRNA targeted GFP mRNA. Firstly, we checked the viability of this siRNA. Results from fluorescence and Western blot showed that GFP were knocked down by pSuper-siGFP clearly when plasmid encoding GFP was transfected with pSuper-siGFP (Figure 2C). Then this siRNA was used to knock down M mRNA in HPIV3_MGFP_ infected cells. The transcription level of M-GFP were examined by RT-PCR to confirm the effect of the GFP targeted siRNA in HPIV3_MGFP_ infected cells. As showed in Figure 2D, the results of RT-PCR showed that the transcription levels of M and GFP were sharply decreased with increasing amounts of pSuper-siGFP, suggesting that the siRNA targeting GFP decreased the transcription of M. Meanwhile, the results of Western blot also showed that the expression levels of M decreased gradually with increasing amount of pSuper-siGFP. Viral HN slightly increased in pSuper-siGFP-transfected cells than that of mock-transfected cells. However, viral titer in pSuper-siGFP-transfected cells was decreased dramatically due to the absence of M which was the primary driving force for viral budding (Figure 2D, right panel), suggesting that M inhibited viral replication as well. Next, we sought to know if we could exclude the impact of siRNA on viral replication by expressing M. HeLa cells were transfected with pSuper-siGFP and infected with HPIV3_MGFP_. At 24 hpi, 1 µg of pcDNA3.0 vector or pcDNA3.0-HA-M were transfected. The results in Figure 2E showed that M could counteract the effect of pSuper-siGFP and decrease the expression of HN. Meanwhile, M rescues virus titer when it was transfected. Taken these data together, we concluded that not only the mingenome-encoded reporter activity but also the replication of HPIV3 could be decreased by M.

### 3.3. The Inhibitory Activity of M Is Independent of Its Budding Ability

M of HPIV3 has been shown to release VLPs when it is expressed alone [21]. Viral N and P can be incorporated into the VLPs via M–N and M–P interaction [22]. Therefore, we wanted to evaluate whether the VLP release is responsible for the inhibitory activity of M. For this purpose, two point mutants of M, M_L302A_ and M_L305A_, were examined in the modified minigenome assay. The properties of the two point mutants were showed in Table 1. 

**Figure 3:**
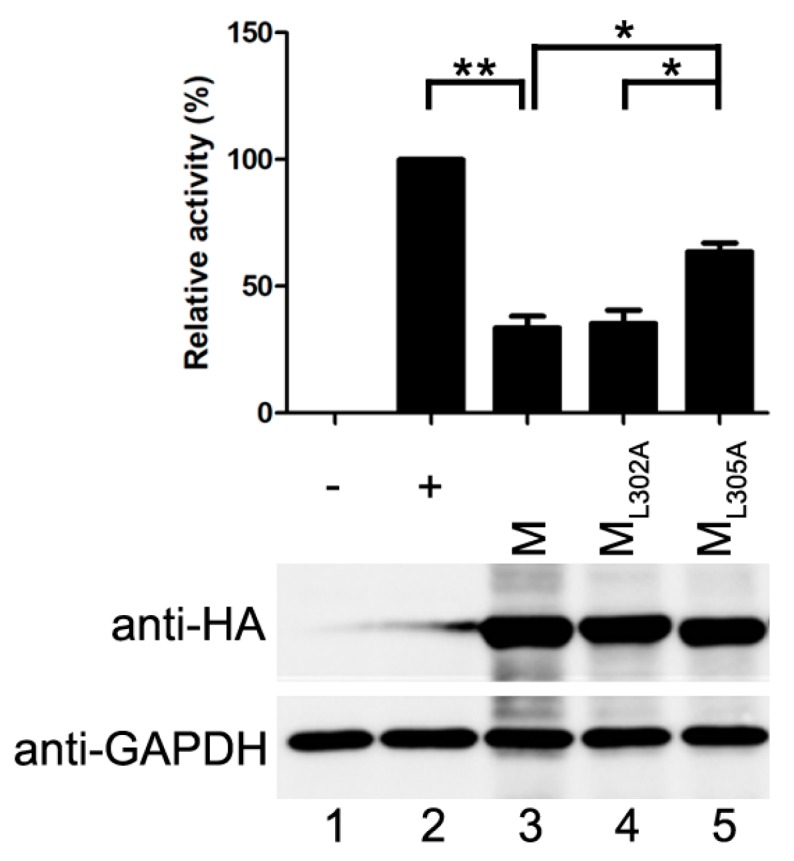
The inhibitory activity of M is independent of its VLP release. vTF7-3-infected HeLa cells were transfected with plasmids encoding N, P, L, minigenome, renilla luciferase and M or M mutants. Relative luciferase activity was measured and the expression of M was detected via Western blot as described for Figure 1C. Values are means ± SD from three experiments. Student’s *t* test: * *p* < 0.05, ** *p* < 0.01. “+,−” means with or without transfecting P plasmid.

The M_L302A_ abolished the ability to release VLPs but maintained associations with N and P [22]. The M_L305A_ has an ability to release VLP and interacts with P as M does, but fails to interact with N [21,22]. When M_L302A_ was transfected with the minigenome assay components, it reduced the reporter activity as M did, and the relative reporter activity dropped to about 35% of that in positive control, suggesting that budding of M has no effect on its inhibitory activity on reporter RNA synthesis. However, when M_L305A_ was transfected into the minigenome system, the relative reporter activity dropped to 64% of that in positive control and all the mutants were expressed at a similar level to M (Figure 3). The results of the minigenome reporter showed that M inhibits reporter RNA synthesis via M–N interaction partly. These data suggest that the budding ability of M has no effect on viral minigenome-encoded reporter RNA synthesis, and M–N interaction might be the main cause for the inhibitory effect of M on the RNA synthesis.

### 3.4. M Reduces the IB Formation via M–N Interaction

**Figure 4:**
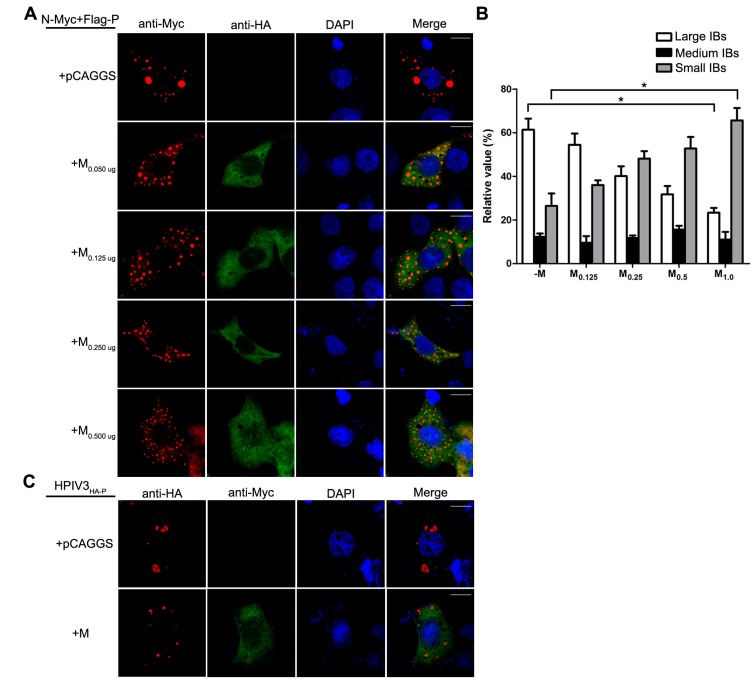

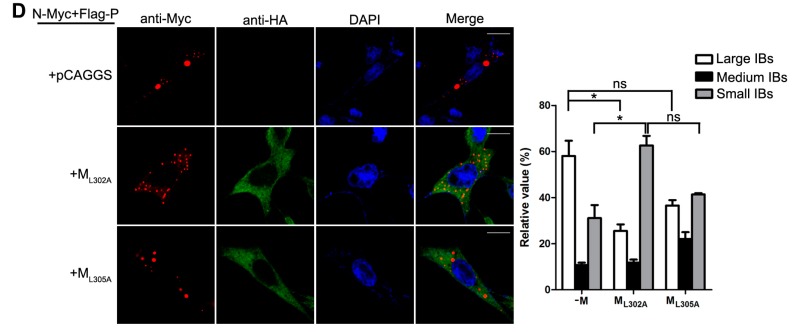
M reduces the formation of IBs. (**A**) the effect of M on IB formation. HeLa cells were transfected with plasmids encoding N and P alone or together with increasing amounts of pCAGGS-HA-M (50, 125, 250, 500 ng). At 24 hpt, cells were fixed and stained with antibodies against Myc and HA and visualized via confocal microscopy as described in Material and Methods. DAPI was used to stain the nuclear; Scale bar, 10 µm. (**B**) the quantifications of large, medium and small IBs relative to IBs of all sizes in M transfected cells. The cross-sectional areas of IBs in (**A**) were measured. Large, medium, and small IBs were quantified in at least 50 cells for each group. The graph shows the percentages of large, medium, and small IBs to IBs of all sizes. Values are means ± standard deviations (SD) from three experiments; Student’s *t* test: * *p* < 0.05. (**C**) M regulates IB formation in HPIV3-infected cells. HeLa cells were transfected with plasmid encoding M. At 24 hpt, cells were infected with HPIV3_HA-P_. At 48 hpi, cells were fixed for immunofluorescence assay as described in (**A**); Scale bar, 10 µm. (**D**) the effect of M mutant on the formation of IBs. HeLa cells were transfected with plasmids encoding N and P alone or together with M_L302A_ or M_L305A_. At 24 hpt, cells were fixed for immunofluorescence assay as described in (**A**). The quantifications of large, medium and small IBs relative to IBs of all sizes were calculated as described in (**B**). Values are means ± standard deviations (SD) from three independent experiments. Student’s *t* test: * *p* < 0.05, ns = not significant. Scale bar, 10 µm. “+” means with transfecting corresponding plasmids in (**A**,**C**,**D**).

Having established that the decrease of viral minigenome-encoded reporter activity was not due to the budding deficiency of M, and IBs are thought to be sites for RNA synthesis [10], and our previous work suggested that HPIV3 N protein and P protein are sufficient to form IBs [9,22], we further explore the relationship between M and IBs. To better validate the effect of M on IBs formation, an immunofluorescence assay was performed by co-expression of N and P with increasing amounts of M. As shown in Figure 4A, large IBs were observed when N and P were co-expressed without M. With the increasing amounts of M, increasing small IBs with a mean cross-sectional area less than 3 μm^2^ appeared. In order to better evaluate the formation of IBs, large, medium and small IBs according to the categories of IBs in Table 2 were counted respectively in at least 50 cells. The percentages of these three kinds of IBs were calculated and presented as a bar graph in Figure 4B. These data clearly suggest that M reduces IBs formation, but it does not co-localize with IBs even though M interacts with N and P, respectively. Furthermore, an immunofluorescence assay was performed in HPIV3 infected cells. The HPIV3 infection was replaced by a recombinant HPIV3 with a HA tag fused to the N-terminus of P that could be detected by antibodies against HA. In HPIV3_HA-P_ infected cells, M reduced the dimension of IBs compared to IBs in M mock-transfected cells (Figure 4C), suggesting that M indeed has an inhibitory effect on the formation of IBs. Similar detections were performed for M_L302A_ and M_L305A_ to evaluate the effects of the two mutants in IBs formation and the percentages of these three kinds of IBs relative to IBs of all sizes were calculated and presented in Figure 4D. M_L302A_ inhibited the IBs formation as M did and M_L305A_ slightly increased the percentages of large IBs and medium IBs compared with that in M (compare Figure 4D with Figure 4B). This result was in agreement with a functional assay in Figure 3. Taken together, our results suggest that M regulates IB formation and inhibits viral replication by interacting with N. 

## 4. Discussion

In this study, we present evidence that HPIV3 M reduces minigenome-encoded reporter activity and HPIV3 replication (Figure 1 and Figure 2). Subsequently, we further demonstrate that the inhibitory activity of M was independent of its VLPs release ability (Figure 3). As IBs are proved to be the sites for viral replication and transcription, we examined the effect of M on IB formation and found that M regulated the formation of IBs (Figure 4A,C). By using M_L305A_, which is defective in M–N interaction, we found that M reduced viral replication via the M–N interaction. The M_L305A_ mutant slightly increased minigenome-encoded reporter activity and the percentage of large IBs compared with the M and M_L302A_ (Figure 3 and Figure 4C,D). 

Several studies have explored the mechanism of M in regulating NNSV RNA synthesis. Different viruses utilize different mechanisms. Some NNSVs M shifts the balance between transcription and replication [24]. M of some NNSVs regulates the formation of nucleocapsid [23]. HPIV3 M regulates viral replication and IB formation via M–N interaction. These findings reflect the different properties of NNSVs M. M of EBOV and Borna disease virus have been shown to bind to RNA [28,29,30], which is essential for the life cycle of EBOV even though the identity of RNA was not clear. However, it is currently unclear in our study whether HPIV3 M binds to virus RNA and the RNA binding activity of M is one of the reasons for its inhibitory effect on viral RNA synthesis. After all, M_L305A_ deprived of the M–N interaction still decreases the minigenome reporter activity to 64% of that in M mock-transfected control, implying that there may be some other reasons involved in the RNA synthesis inhibition of M. In addition, the M of Tacaribe virus inhibits viral RNA synthesis in minigenome assay via interacting with viral L [31], and we do not know whether HPIV3 M interacts with viral L and whether M–L interaction is another reason for the inhibitory effect of M on minigenome-encoded reporter. Additional experiments are needed to address these questions.

HPIV3 M was found to reduce the expression of cellular genes or T7-promoter driven genes. When M was co-expressed with the T7-promoter driven renilla luciferase, it reduced the renilla luciferase activity (Figure 1B). A similar effect of M has been found in other NNSVs such as the M of VSV [32], VP40 of EBOV and MARV [26,33]. To analyze the extent to which this effect on T7-promoter driven genes expression is responsible for the inhibition of HPIV3 replicaiton, a T7-promoter driven renilla luciferase was also added into the minigenome system to reflect the expression of T7-promoter-driven minigenome assay components. By calculating the ratio of minigenome encoded firefly luciferase activity to renilla luciferase activity, suggesting that M of HPIV3 has an additional, direct effect on minigenome-encoded reporter RNA synthesis in spite of its side-effect on the expression of T7-promoter-driven minigenome assay components. The expression of the minigenome reporter actually includes the transcription of the minigenome RNA and replication minigenome RNA. We tried to distinguish the effect of M on minigenome transcription and replication, respectively, by constructing a replication-defective minigenome with the trailer sequences deleted. Unfortunately, this ΔTr-minigenome did not work at all. Thus, we can not distinguish the effect of M on minigenome transcription with replication. 

M of NNSVs are a multifunctional protein, which play a key role in determining morphology of virions by directing viral assembly and budding. In addition, over-expression of M protein will also inhibit viral RNA synthesis and reduce the accumulation of viral N, P, L protein and viral genome. To some extent, over-expression of viral M is harmful for viral assembly and budding even if M is the main force for viral assembly and budding. In HPIV3 infected cells, the expression of M was strictly regulated and the virus must find a balance to regulate the expression of M somehow. Therefore, the effect of M in inhibiting viral protein synthesis and viral assembly and budding will be modified. However, in our assay, the effect of exogenetic M has been examined extensively. As shown in Figure 2, even though the effect is limited, the inhibition of M in viral protein synthesis and viral titer exists indeed.

Previous studies have shown that the release of recombinant rabies viruses and measles viruses that lack M is drastically impaired [11,12]. When we used pSuper-siGFP to knockdown the expression of M, M will exert a greater effect on inhibiting RNA synthesis in a single cell. Therefore, the expression of viral HN protein should increase in a single cell once knockdown of M has occurred. However, lacking M will hinder viral assembly and budding, thus, resulting in decrease of the newly infected cells. We could draw this conclusion from titer assay shown in Figure 4D, right panel. Taken all of the cells as a whole, viral HN protein should decrease, which showed a negative effect finally. When we examined the expression of viral HN protein by Western blot, we collected all of the cells for our assay. The positive effect of M will be counteracted partly by the negative effect. Therefore, the effect of the silencing strategy on HN protein expression is limited in Western blot. We repeated this experiment several times and found that, when we knocked down M protein, the expression of viral HN protein increased slightly in a dose dependent manner.

Although M of HPIV3 has an ability to release VLP, the VLP release ability has no effect on viral RNA synthesis. The M_L302A_, which is destroyed the VLP release ability, did not increase the activity of minigenome encoded reporter (Figure 3). In contrast, the M_L305A_ that is defective in M–N association decreases the activity of minigenome slightly (Figure 3) and increases the percentages of large IBs compared with that of M (Figure 4D). Furthermore, in our pervious study, we found that HPIV3 with M_L305A_ in the genome replicated more slowly than the HPIV3 [22]. The underlying mechanisms may partially lie in the inhibition of IB formation since viral RNA synthesis occurs in the IBs. However, more evidence should be provided to confirm this hypothesis. These data suggest that M reduces viral replication and the formation of IBs via the M–N interaction. It seems that N is a main target for M to inhibit viral RNA synthesis as it has been reported that MeV M inhibits viral RNA synthesis only when it is able to interact with the N [25]. We wonder whether M–P is another reason for the inhibition of M since M also interacts with P. However, we failed to find a mutant that is defective in M–P interaction, it is hard to assess the effect of M–P interaction on the RNA synthesis inhibition of M. However, M does not co-localize with IBs even though it interacts with N and P and decreases the dimension of IBs. These results suggest that the association of M–N and M–P may be indirect, and there may be some cell factors involved in the M–N and M–P interaction and it will be a subject for future study. Meanwhile, we cannot exclude the possibility that the interaction between N and P blocks the sites for M–N and M–P interaction so that the co-localization of M and IBs cannot be detected. In our previous study, the C-terminal of P demonstrated being involved in the association with N and M [22]. N binds to the C-terminal of P and prevents the interaction of M–P. In addition, in IB formation, small IBs were found to fuse with each other to form large IBs via acetylated α-tubulin [10]. Our latest work shows that large IBs have a trend to dismiss at the later stage of transfection. In this study, we found that the IBs were diffusely distributed throughout the cells when N, P and M were co-expressed. We could not confirm whether M inhibits the formation of IBs or accelerates detachment of IBs, and this will be an important issue for future studies to explore possible candidates of cell factors involved in M–N or M–P interaction and reveal the inhibition mechanism of M during the course of a natural infection.

## 5. Conclusions

In summary, our results demonstrate that HPIV3 M inhibits viral replication, which is independent of its VLP formation function. We propose a model in which M reduces HPIV3 replication and IB formation via M–N interaction. To our knowledge, our results provide the first detailed experimental characterization of the IB formation and the inhibition of M as a valuable target for rational antiviral approaches. 

## Figures and Tables

**Table 1:** Properties of M and M mutants [21,22].

Protein	VLP Formation	M–N Interaction	M–P Interaction
M	+	+	+
M_L302A_	-	+	+
M_L305A_	+	-	+

+: Keep functional.

**Table 2:** Category of IB according to its size.

Category	Size (Mean Cross-Sectional Area)
Large	Greater than 8 μm^2^
Medium	Between 3 μm^2^ and 8 μm^2^
Small	Less than 3 μm^2^

## Abbreviations

HPIV3	human parainfluenza virus type 3
M	matrix protein
VLP	virus like particle
N	nucleoprotein
IBs	inclusion bodies
NNSV	non-segmented, negative-strand RNA virus
P	phosphoprotein
L	RNA-dependent RNA polymerase
F	fusion protein
HN	hemagglutinin-neuraminidase
RNP	ribonucleoprotein
GS	gene start
GE	gene end
GJ	gene junction
VSV	vesicular stomatitis virus
RSV	respiratory syncytial virus
NiV	Nipah virus
EBOV	Ebola virus
MeV	measles virus
PIV5	parainfluenza virus type 5
IFN-I	type-I interferon
RABV	rabies virus
MARV	Marburg virus
MOI	multiplicity of infection
DMEM	Dulbecco’s modified Eagle’s medium
FBS	fetal bovine serum
vTF7-3	Recombinant vaccinia virus
ORF	Open reading frame
GAPDH	Glyceraldehyde-3-phosphate dehydrogenase
